# The Influence of Pressing Methods on the Change of Vertical Dimension of Occlusion in Full Dentures

**DOI:** 10.1055/s-0045-1809313

**Published:** 2025-05-30

**Authors:** Alexandre Luiz Carvalho de Oliveira, Kusai Baroudi, Musab Hamed Saeed, Francisco Carlos dos Santos Reis, Milton Edson Miranda, Rafael Pino Vitti, William Cunha Brandt

**Affiliations:** 1 Faculdade São Leopoldo Mandic, Instituto de Pesquisas São Leopoldo Mandic, Campinas, São Paulo, Brazil; 2 Department of Clinical Sciences, College of Dentistry, Ajman University, Ajman, United Arab Emirates; 3 Centre of Medical and Bio-allied Health Sciences Research, Ajman, United Arab Emirates; 4 School of Dentistry, University of Taubaté, Taubaté, São Paulo, Brazil; 5 Department of Implantology, School of Dentistry, University Santo Amaro, São Paulo, Brazil

**Keywords:** vertical occlusion dimension, acrylic resin, microwave system technique

## Abstract

**Objective:**

The objective of this study was to evaluate the alteration of the vertical dimension of occlusion (VDO) of full dentures when different pressing methods are used during the laboratory procedure.

**Materials and Methods:**

Sixty specimens were divided into four groups ( *n* = 15). In all cases, as an antagonist model, a standard assembly with cast alloy teeth was used, all the top pressed models were mounted in semiadjustable articulator through a metal reassembly plate. The vertical distance evaluation was performed by vertical measurement, using a digital caliper. The groups were divided as follows: group 1 (the muffle was not opened, and the space propitiator was not used), group 2 (the muffle was opened and the space propitiator was not used), group 3 (the muffle was not opened and the space propitiator was used), and group 4 (the muffle was opened and the space propitiator was used). In the four groups inclusions were carried out with zero expansion gypsum, 1000 kgf press, Vipi STG muffle and Vipi thermopolymerizable resin. The four groups were polymerized by the microwave power system with 800 watts of power.

**Statistical Analysis:**

Statistical analysis was performed using one-way analysis of variance and Tukey's *post hoc* test ( *α* = 0.05).

**Results:**

Groups 3 and 4 presented the smallest change in the vertical occlusion dimension.

**Discussion:**

Given the methodology applied in this study, the space propitiator was able to act significantly in reducing the change in VDO. There are many factors related to dimensional changes in the bases of full dentures. The dimensional changes and its relationship with the movements of artificial teeth have been observed. Some studies have shown that fixing or not fixing the artificial teeth before pressing does not prevent tooth displacement during subsequent polymerization and flasking stages.

**Conclusion:**

The use of the space provider was effective in decreasing the vertical occlusion dimension, while the opening of the muffle for removal of the excess acrylic resin proved to be dispensable.

## Introduction

Since the development in dentistry, in 1936, of the acrylic resin based on methyl methacrylate, it has been a challenge for dental surgeons to control the consequences of its alterations in the manufacture of the base of total prostheses. 1 2 3 The change in vertical dimension of occlusion (VDO) resulting from the polymerization of full dentures may affect the condylar and occlusal relationships of patients, tension in the facial muscles, and noise from the dentures during speech or even an aged facial appearance. 4 5

Goodacre et al reported that after the prosthesis is polymerized there are changes in the laboratory process with the incorporation of errors, which can change the VDO. 6 7 8 9 Thus, it is necessary to have a rigorous control and knowledge of materials in this phase since the VDO should remain unchanged, from the stage of making the occlusion arches until the finalized prosthesis is placed in the patient's mouth.

Compagnoni et al evaluated the influence of the polymerization cycle on the VDO in total prostheses and concluded that regardless of the polymerization cycle used, there were changes in the VDO in total prostheses, with average increases prevailing in all of them. 10 11 Goldstein et al developed research where he evaluated the alteration of the VDO in superior total prostheses and in acrylizations with microwave energy polymerization cycle and concluded that the acrylized superior total prostheses presented a smaller alteration in the VDO. 5

Other changes may also occur 12 13 due to linear contraction of acrylic resin, plaster expansion, incorrect inclusion, and induced tensions, all are factors that induce changes in the vertical dimension by spatial movement of teeth. 1 10 14 15 16 In order to control some of the dimensional changes that occur during the processing of total prostheses, it is interesting to use a technique that proposes an internal relief in the muffle in order to create a space for the acrylic resin to accommodate and be expelled, reducing the formation of internal stresses and allowing the two parts of the muffle to adapt correctly, minimizing the dimensional change of acrylic resin that causes an increase in the VDO. 17 18 19 This study evaluated methodologies of inclusion and polymerization, seeking the results that present less change in the VDO. The aim of this study was to evaluate the change in the VDO of full dentures when different pressing methods were used during the laboratory procedure, with the null hypothesis of not changing the DVO using the space provider.

## Materials and Methods

**Fig. 1:**
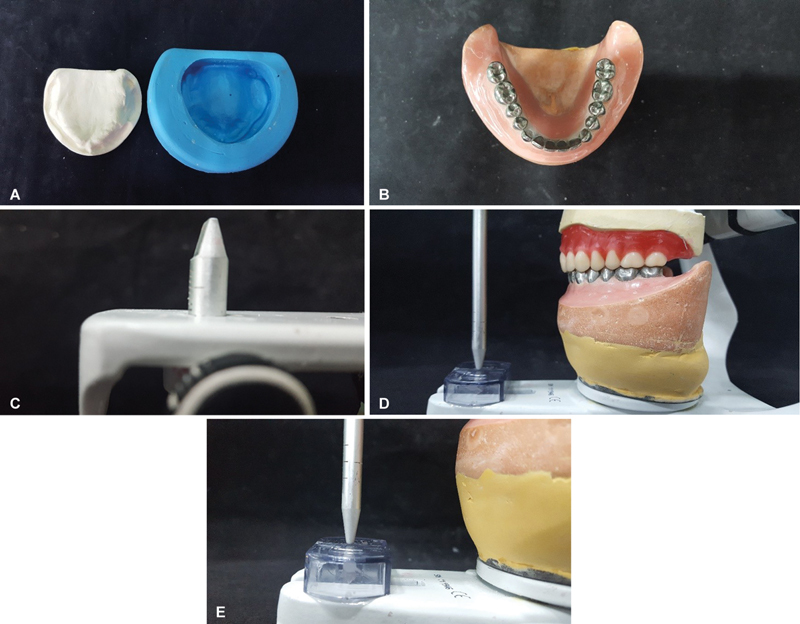
( **A** ) Type IV special plaster master model and industrial silicone mold to obtain the 60 working models. ( **B** ) Lower master model with all teeth cast in metal. ( **C** ) Guide pin in the zero-mark upper model mounted and articulated with the lower model. ( **D** ) Upper model assembled and articulated with lower model. ( **E** ) Guide pin touching the incisal table.

The working models were obtained from a master maxillary model of special plaster type IV zero stone, from this master model a rubber mold was reproduced with industrial silicone and then from this rubber mold 60 models were reproduced with special plaster type IV zero stone ( Fig. 1A ), where 23 mL of distilled water were used for each 100 g of plaster spread in a vacuum spreader (Metal Vander) being 15 models for each group.

The lower mandibular model was obtained using a standard master model, where all teeth are of cast metal structure ( Fig. 1B ).

A double total prosthesis was assembled with the aid of the camper table on an ASA A7 Plus Bio Art, the upper model with Triunfo brand stock teeth, models 264, 34L, in color 62, antagonizing with a standard mandibular lower model, where all teeth were of cast metal structure, and the guide pin was kept at the zero demarcation, touching the center of the incisal table ( Fig. 1C–E ).

**Fig. 2:**
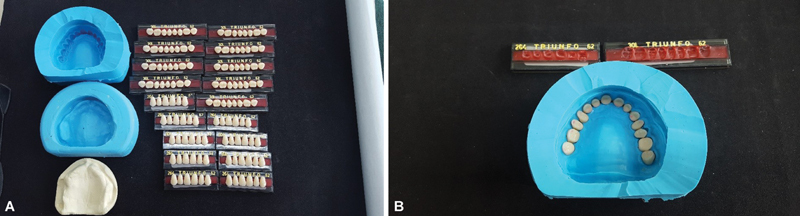
( **A** ) Duplicate industrial silicone mold—master model and master assembly. Standard model/rubber assembly and standard assembly. ( **B** ) Placing the artificial teeth in the obtained mold.

From this assembly, another copy was made with industrial silicone rubber for molds Siqmol 6008 (Siquiplas, São Paulo, Brazil), in order to have the reproduction and standardization of the model and maxillary assembly set. Next, the stock teeth were positioned in the “master mold” to obtain the 60 standardized assemblies ( Fig. 2A and B ).

**Fig. 3:**
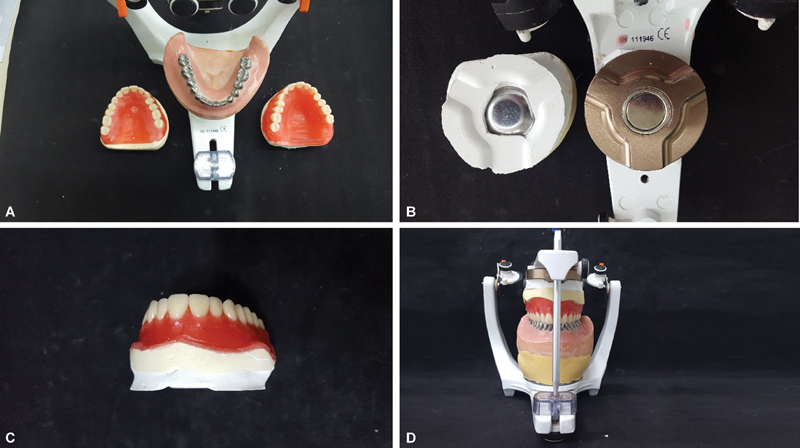
( **A** ) Mounted and duplicated models with lower master model. ( **B** ) “Split cast” set. ( **C** ) Model duplicated with plaster/magnet. ( **D** ) Master mounted upper model with the split-cast-magnet assembly together, with the guide pin demarcated at zero and touching the incisal table.

Using the standard model mount, 60 replicas of the standard mount were reproduced, and all these mounts were mounted in ASA ( Fig. 3A–D ) at the same height as the incisal guide pin at zero and with the split-cast/magnet-plaster assembly.

After assembling the teeth, the inclusions were divided into four different groups. All 60 prostheses were included together with the plaster-magnet base, relieved with Labor Mass Condensation Silicone (Vipi Produtos Odontológicos Ltda, Pirassununga, São Paulo, Brazil) for laboratory use, and isolated with VIPI FILM Acrylic Resin Insulator (VIPI Produtos Odontológicos Ltda). All 60 inclusions in the muffle base were standardized in the same way, using a microwave muffle, Zero Arti (Dentona AG - Otto-Hahn-Str.27.44227, Dortmund, Germany) and zero-expansion stone plaster (Dentona AG - Otto-Hahn-Str.27.44227) spatulated in a Metal Vander vacuum spatulator (Metal Vander, Piracicaba, São Paulo, Brazil).

The models were divided into four groups with 15 models each, with two different inclusion methods in the counter-muffle, as shown in Table 1 .

**Fig. 4:**
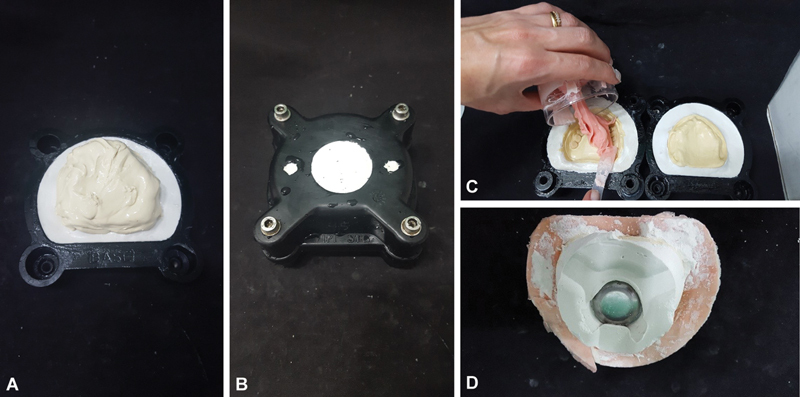
( **A** ) Type IV Zero Stone plaster wall in group 1. ( **B** ) Completed inclusion of group 1. ( **C** ) Denture of the resin for pressing. ( **D** ) Prosthesis noninclusion and preserved magnet/plaster set.

**Fig. 5:**
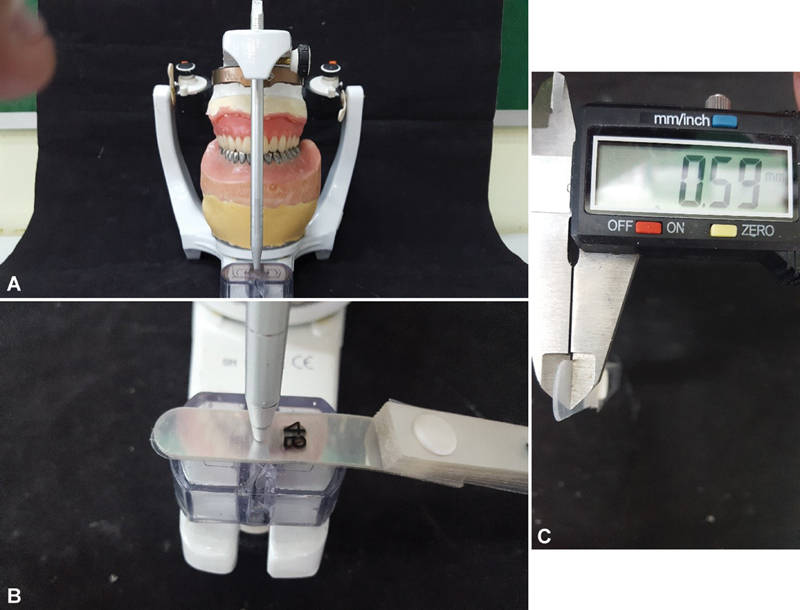
( **A** ) Reassembling the prosthesis in the ASA and distance between the guide pin and incisal table. ( **B** ) Filling of the distance with polyester strips. ( **C** ) Measuring with caliper the distance obtained with the polyester strips.

**Fig. 6:**
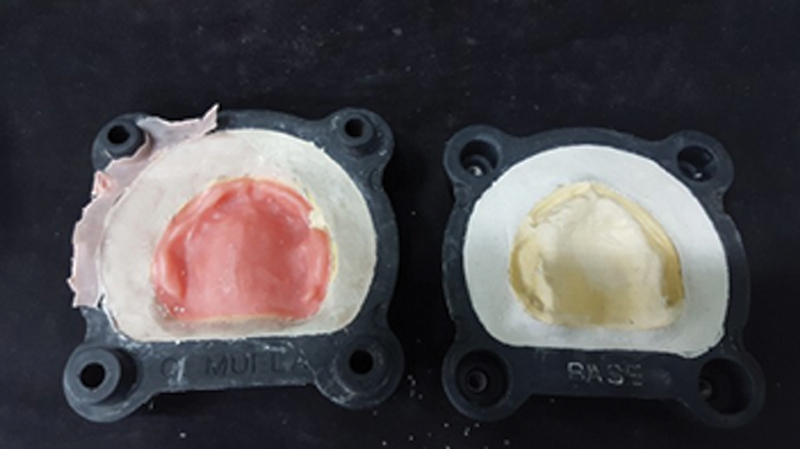
Opening of the muffle after 3 hours.

**Fig. 7:**
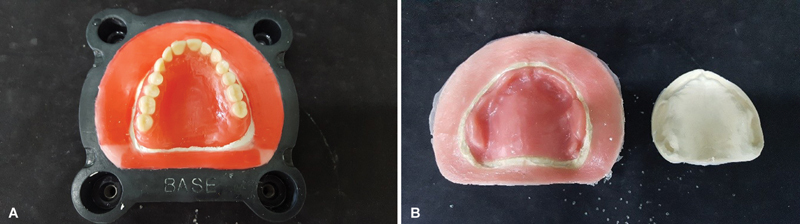
( **A** ) Space promoter made with wax 7, before making the stone plaster wall. ( **B** ) Excess resin layer after opening the muffle.

**Fig. 8:**
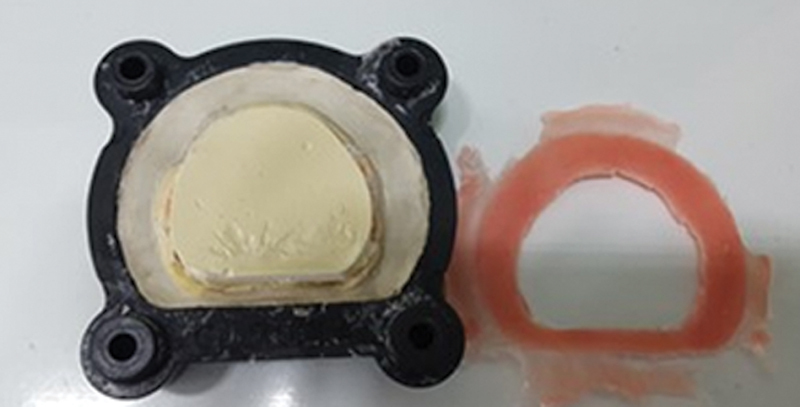
Opening of the muffle after 3 hours of pressing and removal of excess resin.

- Group 1 (the muffle was not opened, and the space maintainer was not used)
In this first group no inclusion with space builder was performed. After crystallization and smoothing of the plaster at the base of the muffle, the common plaster at the base was isolated with pasty Vaseline and then a type IV Zero Stone plaster wall (Dentona AG - Otto-Hahn-Str.27.44227) was made, and then the rest of the counter-muffle was completed with the same plaster ( Fig. 4A–D ).
  After crystallization of the plaster in the counter-muffle, the muffles were opened, washed with hot water, degreased with detergent, and insulated with acrylic resin insulators, and polymerized with Vipi Cril Plus (Vipi Produtos Odontológicos Ltda) thermopolymerizable acrylic resin.
  The acrylic resin was pressed with the muffle at 1,000 kg/f in a hydraulic press (Vipi Produtos Odontológicos Ltda), the muffle was not opened, and the resin “rested” in the press for a period of 12 hours. Then, the muffle was hydrated for 10 minutes in water and polymerized by the microwave system technique.
  An Eletroclux microwave oven (800–1,200 watts model) was used and polymerized at 30 w for 10 minutes, 0 power for 10 minutes, and 40 power for 10 minutes. The cooling was natural and the demoulding was done with the aid of a pneumatic demoulder, preserving the model with the magnet/plaster set, which will be returned to the articulator. This polymerization protocol was used in all four groups.
The prosthesis was reassembled on the ASA using the magnet/plaster/split-cast system ( Fig. 5A ). The measurement of the VDO variation, which occurred in all groups, was measured and standardized for the four groups as follows:
The distance from the guide pin clearance to the incisal table was filled using 0.1 mm thick polyester strips (Leaf Gauge) until they touched on both sides ( Fig. 5B ). The strips were removed and with a Mitutoyo Digital Pachymeter the VDO variation was measured ( Fig. 5C ).
- Group 2 (the muffle was opened, and the space builder was not used)
For group 2, no inclusion with space builder was performed. After crystallization and smoothing of the plaster at the base of the muffle, the common plaster at the base was isolated with pasty Vaseline, and then a Zero Stone (Dentona AG - Otto-Hahn-Str.27.44227) type IV plaster wall was made, and then the rest of the counter muffle was completed with the same plaster. Different from group 1, after carving and pressing at 1,000 kg/f, the muffle was opened after 3 hours of pressing ( Fig. 6 ), where a large film of excess resin also formed, this film was removed, and the prosthesis was pressed for another 12 hours (rest time). The polymerization and laboratory reassembly occurred following the pattern adopted in group 1.
- Group 3 (the muffle was open, and the space improver was not used)
For group 3, the space builder was used, which according to the methodology described by Nogueira, 19 consists in applying a 2-mm thick layer of wax to the plaster on the base of the muffle, within 3 mm around the model ( Fig. 7A ). The counter-muffle is filled and after the removal of the wax, a space will remain to accommodate the excess acrylic resin without moving the edges of the muffle, reducing the formation of internal tensions to the mass caused by the different coefficients of thermal expansion of the plaster, resin, and muffle.
Then, the wall was made with zero expansion stone plaster type IV and the complement of the muffle with the same plaster equally in groups 1 and 2. Pressing of the muffle with the acrylic resin at 1,000 kg/f in a hydraulic press, the muffle was not opened and was left resting in the press for a period of 12 hours, and then the muffle was hydrated for 10 minutes in water and polymerized by the microwave system technique. The formation of an excess resin layer was noted ( Fig. 7B ). The measurements of the variations were also performed equally to groups 1 and 2.
- Group 4 (the muffle was opened, and the space builder was used)
For group 4, until the acrylic resin pressing stage, the same steps were followed as for group 3, with the use of the space builder and, similarly to group 2, opening the muffle after 3 hours of pressing to remove the excess resin that was formed in the space created by the space builder wax ( Fig. 8 ). Again, the muffle was closed and left resting in the press for another 12-hour period, and then the muffle was hydrated for 10 minutes in water and polymerized by the microwave system technique. The results were also measured according to groups 1, 2, and 3.
Prior to the analyses, the data of change in VDO of the total prostheses were evaluated for normality by the Kolmogorov–Smirnov test. Then, they were submitted to one-way analysis of variance. The factor of the study was the pressing method used. Multiple comparisons were performed by Tukey's test. Statistical calculations were conducted adopting the significance level of 5% ( *α* = 0.05), in the SigmaStat 3.5 program (Systat Software Inc., San Jose, California, United States).

**Table 1:** Methods of inclusion used

	Use of space builder	Opening of the muffle in the borrachoid	Phase 12 hour rest pressed at 1,000 kg/f
Group 1 (G1)	No	No	Yes
Group 2 (G2)	No	Yes	Yes
Group 3 (G3)	Yes	No	Yes
Group 4 (G4)	Yes	Yes	Yes

## Results

Table 2 shows the mean values and standard deviation of the change in VDO of the full dentures.

**Table 2:** Mean and standard deviation (millimeters - mm) of the change in vertical occlusion dimension (VDO) of full dentures

Group	Space maintainer	Muffle opened	VDO
G1	No	No	1.70 (0.39) A
G2	No	Yes	1.09 (0.14) A
G3	Yes	No	0.61 (0.18) B
G4	Yes	Yes	0.21 (0.10) B

Note: Averages followed by distinct letters indicate significant difference.

The two-way analysis of variance showed that there was a statistically significant interaction between the factors studied ( *p* < 0.001).

According to Tukey's test, groups G1 and G2 showed the highest values of change in VDO, showing no statistically significant difference between them. Groups G3 and G4 showed the lowest values of change in VDO and showed no statistically significant difference between them.

## Discussion

Considering the increase in VDO caused by the dimensional changes of acrylic resins, it is important to evaluate these alterations in complete dentures. After the polymerization of the acrylic resin is complete—but before the prosthesis is removed from the model - it should be repositioned in its original location on the articulator. This allows for the occlusion to be rechecked and, if necessary, corrected through adjustments. 20 21

Polymethylmethacrylate is the most common resin used in the manufacture of total prostheses, and since its appearance there has been a continuous search to improve its physical and mechanical properties. 15 22 The technical evolution achieved was not enough to totally dominate its alterations, and several techniques have been modified and introduced in order to minimize such alterations. 13

According to several authors, numerous researches have been performed with the purpose of controlling the dimensional changes in relation to the increase in the VDO. 2 13 20 23 Given the methodology applied in this study, the space propitiator was able to act significantly in reducing the change in VDO.

There are many factors related to dimensional changes in the bases of full dentures. Numerous authors observed the dimensional changes and its relationship with the movements of artificial teeth. 1 10 15 16 22 Some studies have shown even with the fixation or not of the artificial teeth, before pressing, it does not prevent tooth movement during the following stages of polymerization and noninclusion. 10 15 16

According to Consani et al, the insertion phase of the acrylic resin is one of the relevant factors in the dimensional change of the prosthesis base and consequently increase in the VDO, concluding that the plastic phase was the one that provided less misfit 13 20 ; however, Turano et al 24 and Gomez et al 25 do not agree with this result, stating that the plastic phase makes an excessive pressure in the pressing, modifying the position of the teeth.

Differently from the results found in this research, the acrylic resin was not pressed in the plastic phase, agreeing with Turano et al 24 and Gomez et al. 25 The opening of the muffle when done, for once, without the propitiator as shown in group 2, had a small decrease in the VDO compared to group 1. When the muffle opening was associated with the space opener as shown in group 4, there was a decrease compared to group 3. Even if statistically the difference between group 4 and group 3 is not significant, opening the muffle is necessary because it helps to reduce internal stresses in the acrylic mass, as reported by several authors. 14 25 26

In this research, the muffle was opened only once, but it would have been possible to open it more times for the removal of excesses, which would facilitate a better adaptation of the muffle and counter-muffle. However, this did not occurred, due to the risk of fatigue of the materials involved due to the multiple pressings. Therefore, these results agree with variables of VDO found by Mahler (1951) 28 , which was on average 0.6 mm, as well as Woelfel et al 27 , which alternated from 0.00 to 1.49 mm, Dukes et al (1983) 29 who found variables of 1.57 mm on average, of which 0.55 mm was base and 1.02 mm on account of dental change, in addition to Savabi et al, who found changes ranging from 0.03 to 1.02 mm. 28

## Conclusion

According to the methodology applied and the results obtained, it can be concluded that the space propitiator technique is effective and contributed significantly in reducing the change in the VDO of full dentures. Furthermore, opening the muffle once to remove excess acrylic resin before polymerization was not effective in controlling changes in the VDO of full dentures.
